# Transforming Ocean Conservation: Applying the Genetic Rescue Toolkit

**DOI:** 10.3390/genes11020209

**Published:** 2020-02-18

**Authors:** Ben J. Novak, Devaughn Fraser, Thomas H. Maloney

**Affiliations:** 1 Revive & Restore, 1505 Bridgeway #203, Sausalito, CA 94965, USA; tomhmaloney@gmail.com; 2 Genetics Research Lab, California Department of Fish and Wildlife, Sacramento, CA 95834, USA; devaughn@reviverestore.org

**Keywords:** genetic rescue, genetic insight, genomic intervention, biodiversity, biotechnology, marine science, marine conservation, ocean genomics, eDNA

## Abstract

Although oceans provide critical ecosystem services and support the most abundant populations on earth, the extent of damage impacting oceans and the diversity of strategies to protect them is disconcertingly, and disproportionately, understudied. While conventional modes of conservation have made strides in mitigating impacts of human activities on ocean ecosystems, those strategies alone cannot completely stem the tide of mounting threats. Biotechnology and genomic research should be harnessed and developed within conservation frameworks to foster the persistence of viable ocean ecosystems. This document distills the results of a targeted survey, the Ocean Genomics Horizon Scan, which assessed opportunities to bring novel genetic rescue tools to marine conservation. From this Horizon Scan, we have identified how novel approaches from synthetic biology and genomics can alleviate major marine threats. While ethical frameworks for biotechnological interventions are necessary for effective and responsible practice, here we primarily assessed technological and social factors directly affecting technical development and deployment of biotechnology interventions for marine conservation. Genetic insight can greatly enhance established conservation methods, but the severity of many threats may demand genomic intervention. While intervention is controversial, for many marine areas the cost of inaction is too high to allow controversy to be a barrier to conserving viable ecosystems. Here, we offer a set of recommendations for engagement and program development to deploy genetic rescue safely and responsibly.

## 1. Introduction

Wildlife biodiversity and abundance are experiencing unprecedented declines, with every marine ecosystem subject to multiple anthropogenic threats [1]. The same primary threats (habitat loss, overexploitation, pollution, invasive species, and climate change) threatening terrestrial and freshwater ecosystems are also affecting oceans, yet the extent of damage impacting oceans and the diversity of strategies to protect it is disconcertingly, and disproportionately, understudied considering the capacity of the ocean to support life.

Human-caused biodiversity loss is significantly outpacing rates of evolution and adaptation in natural populations [2,3]. Under such rampant biodiversity loss, genetic variation that took millions of years to evolve is disappearing, and with it, the potential for remaining populations to adapt to rapidly changing environments [4]. Numerous models show human activity has a disproportionate effect on highly productive coastal areas and regions of high biodiversity [5] and the evidence of plastics present in Mariana Trench invertebrates [6] further demonstrate the alarming extent of human-mediated effects on ocean health. The entirety of life on the planet is dependent, directly and indirectly, on the integrity of ocean health [7]. Millions of human livelihoods depend directly on ocean biota [8]. The ocean is the critical source of globally vital ecosystem services and home to the most bio-abundant populations of life on earth: From the most abundant bacteria, *Pelagibacter*, estimated at 10^28^ cells, and the most abundant phototroph, *Prochlorococcus,* numbering 10^27^ cells [9], to the most numerous animal species, such as the bristletooth fish, numbering 10^18^ individuals [10], Antarctic krill, numbering 10^14^ individuals [11], and copepods, which are innumerable [12]. Therefore, the long-term implications of diminishing ocean health on global biodiversity and human wellbeing likely far exceed (and exacerbate) parallel effects in other ecosystems. 

Over the next 30 years, the human population is expected to grow by 3 billion and per capita resource consumption is also expected to rise [13]. Such precipitous growth increases the urgency to identify radically new methods to maintain Earth’s ecological health. While conventional conservation measures such as minimizing and eliminating pollution, limiting fishing pressure, the interdiction of illegal wildlife trade, and establishment of MPAs are important strategies that help stem the severity of marine threats [2,3,14], these strategies alone cannot completely stem the tide of environmental threats [2,3,15]. The pace and scale of these threats demand immediate innovation in how they are handled. Rapid advances in genomics and biotechnology can provide the technological basis for innovations to complement and potentially transform marine conservation strategies and provide a means for marine systems to persist and evolve [2,3].

Since the first human genome was sequenced in 2003, medicine has been revolutionized by genomic research and its clinical application [16,17,18,19]. In the same period, the integration of biotechnology and genomics into agriculture, while often controversial, has produced novel crop lines that require less land, feed inputs, or pesticides (i.e., [20]). 

Yet the integrated adaptation of genomic tools for wild ecosystems—to support biodiversity, combat disease, or create synthetic alternatives—remains nascent. Exploration of genomics as an innovative strategy to complement and support ocean conservation may provide new wholly methods for combating threats to ocean health. Advances in biotechnology and genomic research should be harnessed and developed within a wide variety of conservation frameworks to foster viable ocean ecosystems far into the future. 

Here, we distill the results of a targeted survey, titled the Ocean Genomics Horizon Scan (OGHS) ([21] available at https://ocean.reviverestore.org), which assessed the opportunities to bring novel genomic and biotechnology insight and innovation to marine conservation. The authors of this paper were part of the survey and assessment team, and undertook an extensive effort that included reviewing current research and conducting interviews with over one hundred ocean science researchers, conservation managers, biotech innovators, and ethicists. From this, we identified how novel approaches from synthetic biology and genomics might alleviate major marine threats. As an assessment of current efforts, we focus on areas where genomics is already being applied or considered, including corals, invasive species, pollution, and overexploitation. Climate change is addressed as a broader and synergistic threat that compounds and exacerbates other marine threats. 

**Figure 1:**
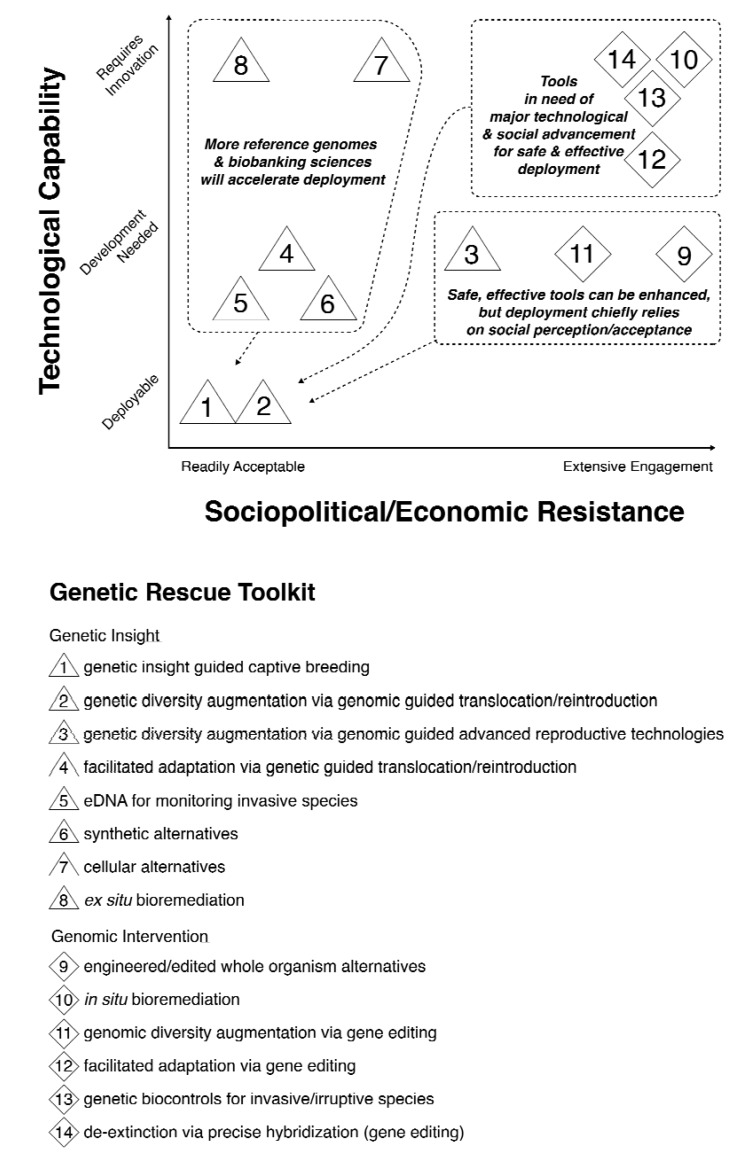
The Genetic Rescue Toolkit Continuum of “Readiness”. A qualitative framework for assessing the technological and sociopolitical/economic factors influencing the readiness and deployment of applications within the Genetic Rescue Toolkit. Triangles denote genetic insight applications and diamonds indicate genomic intervention applications. The sociopolitical/economic axis encompasses consideration of regulations, public perception, cultural influence, and economic factors on the gradient of resistance to use these applications, the left-hand side being acceptable tools and the right-hand being applications met with higher levels of concern. With particular technological developments all these tools can be refined to deployable states, but while social and ethical engagement will improve many tools to acceptable consensus, some tools will face persistent resistance from certain stakeholders for reasons other than safety or efficacy (e.g., ideologies opposing human intervention in nature).

From both technical and social perspectives these innovations are at varying levels of readiness. We developed a qualitative framework for evaluating such innovations and actions, which we call the “continuum of readiness” (Figure 1). Regardless of near-term, long-term, or far-future readiness, a focused initiative to develop these innovations into a holistic Genetic Rescue Toolkit has the potential to help save highly biodiverse marine ecosystems, such as coral reefs. 

Science is just one part of what is necessary to develop and scale these tools to the size of the challenge—social acceptance is another. Many people, from many disciplines across civil society (including scientists, ethicists, conservationists, regulators, and policymakers) must play a role in evaluating genetic rescue tools as a solution set for ocean conservation. Interdisciplinary engagement is the only way to successfully shape the development and suitable application of a marine Genetic Rescue Toolkit. We therefore present our findings on the immediate steps that can be taken to build and apply a dynamic and effective conservation genomics toolkit for marine ecosystems, and in closing, discuss the social, ethical, and economic implications of threats to ocean biodiversity.

## 2. The Ocean Genomics Rescue Toolkit Continuum

In parallel with the Continuum of Readiness for genomic innovations in conservation, a Genetic Rescue Toolkit will necessarily span applications from “genetic insight” to “genomic intervention.” Genetic insight and genomic intervention can accomplish some of the same goals: Maintaining or augmenting genetic diversity and facilitating adaptation to disease or climate change. However, genetic insight, as defined here, primarily uses genetic information (i.e., data derived from genomics, transcriptomics, proteomics, metabalomics, and other collective-omics) to enhance traditional conservation practices. Genomic intervention, as defined here, on the other hand, implements genomic manipulation (engineering or precise editing) to a species of interest and extends the capabilities of genetic rescue further, to include strategies of biocontrol and de-extinction. As such, much of the technology needed for genetic insight applications is ready to deploy for conservation or requires little optimization (Figure 1). Most genomic interventions though, currently require more extensive technological developments before being applied to ecosystems. However, the goal of genetic rescue research and social engagement should be to bring all tools to an effective and safe state of deployable and be readily acceptable.

The following sections outline research areas in which genetic insight and genomic intervention have been demonstrated or are in development to address four major threats to ocean species. 

### 2.1. Overexploitation

#### 2.1.1. Insight for Combating Illegal Trade

Genomics promises to play a major role in improving the traceability of traded and unreported marine wildlife. This requires adaptation of commonly used molecular forensics tools, as well as new innovations. Today, the use of eDNA for wildlife monitoring is diversifying [22], but its application in regulation is still limited. 

Until recently, a major limitation in preventing illegal catch and trade has been the lack of rapid, high throughput technologies. However, new advances that reduce cost and eliminate the need for specialized lab equipment or high-level expertise are promising. The DNA Barcode Scanner by Conservation X Labs for instance, although still in prototype stage, packages the DNA barcoding process into a portable, affordable handheld device, and produces real-time field results [23]. This platform could conceivably be adopted at various checkpoints along the seafood supply chain. Additionally, the pairing of CRISPR technology with alternative proteins (not the Cas9 protein, which is associated with genome manipulation) has the capacity to detect target species with high specificity, yielding detection results within an hour, requiring no DNA extraction, and using just a single reaction tube [24], removing the need for specialized lab equipment and expertise. 

To enhance the efficacy of these approaches, high-resolution genomic data and expanded DNA barcoding repositories are necessary. For example, the accurate genetic discrimination of various mussel species using exceptionally small SNP panels has been demonstrated [25]. These small panels of informative SNPs usually perform better than microsatellite markers when allocating individuals to a geographic origin [26]. Yet SNP panels are largely undeveloped for shellfish due to lack of high-resolution genomic data. 

#### 2.1.2. Insight for Sustainable Fishing

Genetic insight can inform fisheries managers about the impact of fishing on stock vulnerability or the interference of bycatch with restoration efforts (reviewed in Bernatchez et al. [25]). A recent example involves two species of New England herring. Both have experienced major decline, and despite 25 years of conservation effort, recovery has been elusive [27]. However, population genetics using genome-wide microsatellites of offshore fishery samples revealed that >70% of the herring bycatch was from the populations of concern—it is this bycatch that is preventing their recovery [28]. This genetic insight compelled the State of Connecticut to request and receive a 12-nautical-mile closure of the Atlantic herring fishery in September 2018. 

Many cost-effective tools for genetic insight, such as the genome wide microsatellites mentioned above, are enabled by high quality reference genomes. One tool emerging from high throughput genomic tools is the enhanced ability to understand the population dynamics of fisheries through a process known as close kin mark and recapture [29]. These techniques use relatively low cost and high throughput sequencers to uncover kinship relationships within a fishery, through which annual recruitment can be derived. One innovative application of close kin mark and recapture confirmed the ecosystem benefits of coastal California MPAs [30]. 

#### 2.1.3. Insight and Intervention for Alternatives to Wild Harvest

Biotechnology and synthetic molecular biology can also provide alternatives to harvested wildlife products. Several alternatives already exist; the methods by which they were developed could be replicated to alleviate harvest of many other species. These include synthetic recombinant factor C, a replacement for the blood of horseshoe crabs in endotoxin testing by the pharmaceutical industry. The adoption of rFC in drug and water testing not only has the potential to reduce horseshoe crab harvest by >90% but it is also more effective than the lysate tests derived from horseshoe crab blood [31].

While controversial, genetic engineering can reduce overharvesting of a wild product through manipulation of desirable phenotypes. For example, the integration of a pacific salmon growth gene in Atlantic salmon (*Salmo salar*) has produced a faster growing farm population [32,33], reducing the need for wild harvest and ocean aquaculture by vastly improving the efficiency of land-based aquaculture [34]. 

Moreover, in development are plant-based synthesized bioproducts, as well as harvest-free seafood bioproducts manufactured through cell culture [35,36]. Both strategies present an opportunity to address the ecological and economic crisis associated with current seafood fishing and production systems (both wild-caught and farmed) in a scalable and sustainable manner. With major agricultural industry shifts towards eco-agricultural farming practices to relieve the major negative impacts of crop production on biodiversity [37], plant-based meat alternatives can provide synergistic benefits to both terrestrial and marine conservation. 

In contrast to plant-based meat alternatives, cell-culture based meat alternatives are not yet on the market, and may have a long development pathway ahead before attaining the taste, texture, and price that consumers demand. Some companies do aim to launch cellular meat alternatives within 1-2 years, however [38].

Expanding the global database of reference genomes and advancing our understanding of functional genomics is key to expanding the toolkit of wild-harvest alternatives. Specifically, the discovery of new gene variants offers more phenotypic options for improving production of farmed seafood or plant-based alternatives. Understanding the genetic pathways involved in cell differentiation and growth is necessary to establishing commercial scale cell-culture growth platforms and perfecting cell-based meat quality to the level desired by consumers. 

### 2.2. Pollution

#### Insight and Intervention for Plastics Bioremediation

Genomics offers a promising, albeit challenging, approach for the treatment of marine pollution through microbial remediation. Microorganisms, particularly bacteria, can be bioengineered to optimally sense or metabolize contaminants [39]. Genomics, transcriptomics, proteomics, and metabolomics can be applied in a systems biology approach to advance microbial remediation technology [40]. The most promising microbes for effective plastic biodegradation are the *Pseudomonas* species [41]. Recent research has identified *Ideonella sakaiensis* 201-F6 as an efficient biodegrader of PET plastics [42]. Additionally, a recent screen of microbial communities in the Arctic showed promise for plastic degradative abilities in *Rhodococcus* bacterial species and several fungal species [43]. 

Developing adequate bioremediation facilities for plastic can be costly. Once plastic is collected from the ocean, it may require manual processing to sort the plastic into subtypes for different microbes or microbial consortia, and may require pre-treatment to optimize microbial attachment [41]. However, if the engineered microbes are released into the environment and done so safely—and accepted by affected communities—this could greatly increase the scalability of bioremediation by removing the mechanical labor necessary for collecting and transporting pollutants, as well as eliminating the need for bioremediation facilities. 

Substantial time and resources will be required to fully realize bioremediation and synthetic biology as a strategy to combat the ocean plastic problem; however, with well-directed funds, such a strategy could immensely improve conservation outcomes in marine environments.

### 2.3. Invasives

#### 2.3.1. Insight for Monitoring and Early Detection

Genomic technologies, if adequately developed and applied, can also revolutionize our ability to detect invasive species [44]; early detection may enable prevention of their establishment. For example, eDNA can detect a species presence without capture or direct observation, and is currently employed to study invasions in freshwater environments [45,46,47]. Scientists and managers predict that rapid growth, widespread deployment, and automation of eDNA techniques will transform the sensitivity, speed, and scale with which alien species can be detected [48]. Detection of invasive species in ballast water of incoming vessels at ports is an optimal strategic focal point for monitoring efforts [49], and will be enhanced by further advancements in field-deployable sequencing and monitoring tools [24].

Current control efforts of marine invasive and irruptive species primarily involve their manual killing or capture. For example, crown-of-thorns starfish (*Acanthaster planci*) are removed by divers or injected with chemicals. Targeted trapping has shown some promise for European green crab (*Carcinus maenas*) population control [50]. Since European green crabs are edible, there are commercialization efforts underway. Lionfish is also edible and has an emerging commercial harvest, despite the fact that they can be toxic to humans [51]. Killing and capture methods have been successful for small geographic areas, but are expensive and labor-intensive, and are difficult to scale. The high-resolution early detection that eDNA offers may not only improve the efficacy of current control methods, but enable earlier control measures applicable to lower-density populations—reducing the need for more drastic measures against larger, more widespread pest populations. 

#### 2.3.2. Intervention for Genetic Biocontrol

Genetic biocontrol is the release of organisms that have been genetically engineered for the purpose of controlling a pest species [52]. The engineered organism encounters and mates with the wild members of the species and introduces a genetic system that reduces the population size through a variety of mechanisms. An important benefit of genetic biocontrol is the potential to reduce pest species populations while minimizing off-target effects [53]. Genetic biocontrol represents a potentially transformative advance for combatting invasives that is not readily achievable with current technology [54].

There is a varying degree of progress to date with respect to understanding the genomics of the most significant invasive and irruptive species in the ocean. Currently, the crown-of-thorns starfish is a candidate for genetic biocontrol through the use of repressible lethal disruption systems [21]. These systems involve genetic circuits in which immature life stages require the presence of a “repressor” molecule (often tetracycline) that prevents a toxic gene from getting expressed. Mature organisms no longer need the repressor and can be released. Offspring resulting from crosses between wild and engineered organisms die in the absence of the repressor [55].

Other genomic methods of biocontrol include Self-Sorting Incompatible Male/Female System (SSIMS/SSIFS) [56]. These systems engineer a repressible male/female genetic circuit that makes the engineered organism capable of reproducing only with other, similarly engineered, organisms. When a SSIMS organism mates with wild organisms, no offspring survive. However, when a SSIMS male mates with an SSIMS female, only SSIMS males are produced. Researchers modeling SSIMS assert that model results suggest SSIMS has the potential to be more effective than repressible-lethal methods for some species, though more modeling is necessary [56].

Moreover, possible gene drives can be used to spread genes through a population in a way that alters the standard model of inheritance [57]. Normally, releasing an organism with a single copy of a recessive-lethal gene (lethal when two copies are present) will result in the gene’s dilution as it only gets passed on to half of the offspring per reproductive cycle. However, if the recessive lethal gene is part of a gene-drive system, all offspring receive a copy of the recessive gene and it will spread through the entire population. Any mating between carriers results in 100% nonviable offspring (versus 25% under normal inheritance). Gene drive methods have been created in mosquitos as a mechanism to combat malaria [58]. However, the gene drive technique is controversial because of its potential to cause uncontrollable negative effects on ecosystems if the gene drive escapes to nontarget areas, as modeled by population simulations [59]. Other research nullifies this concern and calls into question the efficacy of gene drives because test species have developed resistance mutations [60]. 

Pertaining to ocean ecosystems, gene drives are currently being investigated as a biocontrol method for invasive rodents on islands [61]. Island rodent eradication has been linked to improved coral reef health owing to the restoration of seabird populations following rodent removal; the seabird populations facilitate nutrient transport from terrestrial to marine environments [62]. The primary mechanism to explore the technical and ethical aspects of gene drive in rodent eradication is through the Genetic Biocontrol of Invasive Rodents program (GBIRd). 

### 2.4. Climate Change

#### Insight and Intervention for Adaptation 

Genomic research has already been applied to gathering insight into adaptive variation with respect to climate change. Transcriptome analysis has been used to identify genes involved in climate adaptation in coral [63], and methods to map variation associated with current climate regimes are being developed to model future shifts in species distributions [64]. 

Adaptive interventions would involve the translocation or assisted migration of individuals to match genetic variation within individuals to new environments [65,66,67]. Although the threats due to climate change are widespread, spatial heterogeneity of the ocean and ocean-land interface produces highly localized effects, prohibiting high-resolution modeling to predict climate change impacts. High throughput methods for biosurveying and monitoring local biotic and abiotic parameters are critical in order to adequately and effectively manage adaptive variation in the face of climate change [5]. 

## 3. The Coral Toolkit: A Case Study for Advancing Genomics in Conservation

Coral reef ecosystems are experiencing more frequent and severe bleaching events by changing ocean conditions (warming) from climate change [68,69]. The severity of the threats seems to have engendered a higher level of readiness among coral biologists to consider genomic technologies to facilitate adaptation and resilience to warmer water. As an autogenic ecosystem responsible for 35% of marine biodiversity [70], corals are an exceedingly high conservation priority. 

While coral reefs will regenerate over centuries and millennia through recolonization by thermal tolerant species, the rapid collapse of entire reefs will cause the extinction of thousands of noncoral reef species that have no refuge; these species will be lost completely. It is paramount that intervention can match and overcome the pace of coral reef loss to prevent these extinctions, as well as the major economic losses that will stem from worldwide reef collapse. This pace will only be accomplished if biotechnology and genomics are integrated into intervention strategies.

A major barrier to applying genetic insight and genomic intervention to coral reef rescue is the extreme difficulty in conducting laboratory research on corals because of current difficulties in cryopreserving and maintaining coral colonies ex situ. The following three sections present areas of research that could quickly overcome these technological issues. These and other projects are discussed in more detail in the Ocean Genomics Horizon Scan [21].

### 3.1. Coral Cryopreservation 

Cryopreservation is a vital preventative measure for preserving threatened coral species facing extinction in the wild. Current methods involve the freezing of sperm and larvae harvested from reefs [71,72]. To date, no one has been able to viably freeze and thaw coral eggs. This is a severely limiting factor that requires biologists to mount large-scale, precisely timed expeditions to capture wild eggs at annual spawning events. To expand research opportunities, researchers and restorationists need access to male and female germplasm at any time of the year—not only during natural spawning events. To accomplish this, it is necessary to develop reliable techniques to cryopreserve wild coral eggs. This has led some researchers to examine other cryopreservation methods with coral micro-fragments (pers. comm. Mary Hagedorn).

The micro-fragmentation method offers one approach to scale coral restoration efforts [73,74]. When large fragments of living coral are cut into micro-fragments of ~1 cm^2^, these small clonal pieces grow at exceptional rates to reconstitute larger corals [75]. Micro-fragments can be collected year-round and contain almost all cells necessary for forming adult corals, including symbiotic microbes. Therefore, they represent a more complete sample of coral material than germline or larvae samples. They do not, however, contain gametes. Therefore, micro-fragment banking will be complementary to gamete banking. Using this method may make global collection and preservation of every coral species feasible before oceans reach critical temperature—expected in mere decades.

Multiple groups have developed husbandry methods for making/growing micro-fragments ex situ. The cryopreservation lab at the Smithsonian Institute has identified a suite of cryo-protectants that are nontoxic to micro-fragments and enable them to survive freezing (pers. comm. Mary Hagedorn). The remaining hurdle is to optimize thawing procedures so micro-fragments can grow in open water after years of storage. Once this is achieved, the entire procedure from collection, to freezing and thawing should be standardized and potentially automated. With optimization of cryopreservation techniques, the global collection of coral micro-fragments, sperm, and possibly eggs could soon begin, starting in reefs that are most threatened. Ideally, this coral ark would include 100 individuals of each species, at 10 samples from each individual. No other technology yet offers this depth and continuity for saving a species. 

### 3.2. Inducible Spawning

As mentioned, coral eggs are necessary for breeding but cannot yet be cryopreserved. However, under ideal conditions and proper stimulation, corals can be induced to spawn [76]. Scalable inducible spawning techniques could provide coral breeders a year-round source of eggs, allowing for the efficient use of banked coral sperm.

The Horniman Museum’s Project Coral laboratory is pioneering this work, but scientists there are unable to provide more than one-on-one training at present. By scaling up the team’s laboratory and training capabilities, other practitioners could be trained more easily to perform the inducible spawning technique.

### 3.3. Coral Stem Cells

Ongoing developments in coral stem cell technology and the clonal nature of coral will make it possible to isolate and propagate coral stems cells [21]. Paired with insight from ongoing population genomics, stem cells can be subjected to functional genomic experimentation to determine genotype–phenotype interactions [77]. This work will inform understanding of relative breeding values, and enable appropriate decisions to be made about parental lineages [21].

Stem cells derived from novel breeds could be propagated in a continuous manner in bioproduction facilities to support industrial-scale production of coral larvae for direct out planting of high-performance corals. Moreover, stem cells could be cleaved from juvenile corals, cloned, and then subpooled for genetic experimentation, such that a single coral could be independently tested for many different traits, even with a destructive assay [21]. 

Stem cells with adaptive genes could themselves be transplanted directly into wild corals, in order to recolonize dying coral colonies, as a coral stem cell therapy approach. Finally, by pairing stem cells with genome engineering and gene editing (gene editing has recently been accomplished in a coral species [78]), stem cells could be modified to serve as vehicles to deliver genetic elements for heat resilience into coral reefs. There is growing awareness among coral biologists, as evidenced by the interviewees of the Ocean Genomic Horizon Scan, as well as emerging literature [79], that genomic intervention will play a critical role in conserving coral reefs. 

## 4. The Applicability and Readiness of Genomic and Biotechnological Solutions for Conservation

Throughout this paper, the authors frame the applicability and readiness for biotechnological interventions from the perspective of technological and social factors that will influence development for effective deployment of the genetic rescue tools presented; however, the complete spectrum of factors that will shape how genetic rescue tools are used must include a treatment on bioethics and regulation. Biotechnological interventions for marine conservation will be directly impacted by a number of existing regulatory frameworks, as well as necessitating the incorporation of bioethical analyses to advance to effective and safe states. The reality of regulation is that even for simply building the genome sequence resources needed for genetic insight tools will have to navigate policies impacting every stage of work from tissue collection to database repository and reuse, including the 1972 Convention on International Trade of Endangered Species (CITES), the 1992 Convention on the Conservation of Biological Diversity (CBD), the 2014 Nagoya Protocol, and most pertinent to genomic interventions, the 2000 Cartagena Protocol [21]. These policies were drafted prior to the biotechnological advancements outlined in this paper, and therefore new genetic rescue tools pose certain challenges to regulatory approval and oversight [80]. The Nagoya protocol specifically has hindered taxonomic research [81], the kind that will be crucial to building the shared databases that will make genetic insight tools useful globally. This is not to say that these challenges need to stagnate much needed progress. Modifying policies to reflect the changing environment of conservation needs is ongoing, for example the United Nations has begun drafting policy to rectify the lack of governance of sharing marine genetic resources beyond jurisdictional borders, a gap created by the Nagoya Protocol, under what is being dubbed the “Biodiversity Beyond National Jurisdictions” binding instrument [21,82]. Bioethical considerations are crucial to both intervention practice protocols and their regulating policies [80], but the diversity of ethical and regulatory parameters and ramifications deserve more thorough treatment than can be adequately provided in the context of this paper’s focus. 

Different genomic technologies are at varying states of technological and social readiness for large-scale implementation (Figure 1). Genetic insight can be used to pave the way for more proactive conservation strategies, a shift from the typically reactive state inherent to the “crisis discipline” approach of traditional conservation. Some genetic insight tools require short-term developments for field use (e.g., cryopreservation, cell culture, qPCR, and barcoding) while others, though well developed in other disciplines (biomedicine, agriculture), need more optimization to be applied to conservation efforts (e.g., environmental DNA (eDNA) and CRISPR-assays for monitoring purposes).

Genetic insight can inform conservation management in a variety of ways that are dependent on generating and facilitating access to adequate genomic resources. The exponential increase in genome assemblies and ease of resequencing individuals is establishing the foundations for genetic insight research useful to conservation. For example, cheap and rapid sequencing has enhanced our understanding of population structure for fisheries management [83,84,85], has enabled the characterization and mapping of beneficial genetic variation [86], and has made more rapid identification of novel pathogens associated with wildlife disease possible [87]. High quality reference genomes are particularly important for identifying adaptive variation in natural populations [88]. However, to date, genomics initiatives have never had a specific focus for wildlife conservation, nor have they prioritized marine species (excepting GIGA, Table 1). 

**Figure 2:**
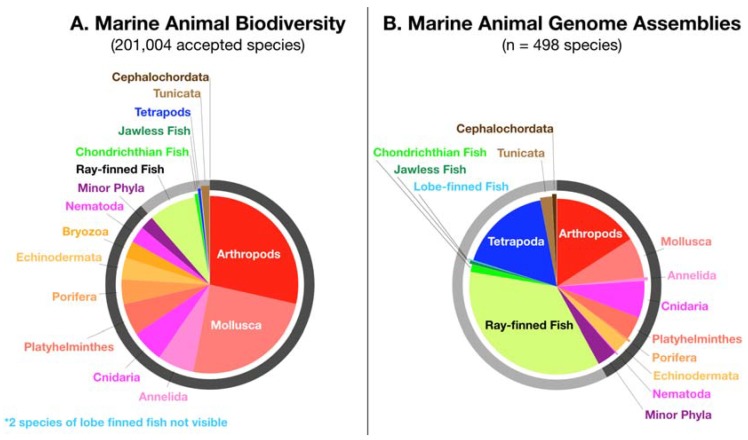
Comparing actual marine animal biodiversity to sequenced marine animal genomes. (**A**) Presents the proportionate taxonomic biodiversity of all accepted marine species [89]. (**B**) Presents the breakdown of marine animal genome assemblies deposited to the National Center for Biotechnology Information as of September 2019. Eleven of the phyla in (**A**) (including one major and ten minor) have yet had a single genome sequenced and assembled. These include Bryozoa, Sipuncula, Nematomorpha, Gnathostomulida, Gastrotricha, Entoprocta, Dicyemida, Cycliophora, Chaetognatha, Aschelminthes. Several of these missing phyla are entirely marine, meaning they are wholly unrepresented in genomics. Outer circles of (**A**,**B**) delineate nonchordate invertebrates (dark gray) from the phyla chordata (light gray), which has been further subdivided, revealing a gross over-representation of sequenced genomes for vertebrate classes compared to entire invertebrate phyla.

The lack of conservation focus in genomics is evident when comparing the ratios of actual marine biodiversity (Figure 2A) to taxonomic representation of genomes deposited to the National Center for Biological Information genomes database (Figure 2B). For a multitude of technical and social reasons, including genome size, ease of tissue preparation, and agricultural and medical relevance, marine genomes are highly skewed to vertebrates, especially fish species (many of which have commercial value). In ocean ecosystems, invertebrates dominate not only bio-abundance, but also a majority of significant ecological roles (i.e., keystone species, ecosystem engineers, foundation species). A database of genomes useful to genetic rescue efforts would more closely reflect taxonomic diversity found in nature (Figure 2A).

In order to enable genetic insight to outpace the decline and loss of species, there is a need for massive investments in a strategic sequencing and biobanking initiative. Such efforts should focus on establishing baseline reference genomes across the ocean “tree of life” in proportion to taxonomic diversity, prioritized by species’ significance to ecological function, and targeted conservation applications. For example, improvement of detection and monitoring of invasives and illegal trade is an urgent priority. However, current eDNA methods can suffer from uncertainties in species identification (especially in marine environments), presenting a risk of false positives, and could have weak statistical power (leading to overconfidence when no detections are recorded) [22,44]. This highlights the need for more high-quality reference genomes of invasive species of concern, as eDNA technologies are likely to become a major focus in invasive species management [48].

Synthetic alternatives are continually being developed, yet adoption at a scale that would impact conservation outcomes are often limited by public preference and pushback from industries that alternatives threaten to replace. For example, the synthetic alternative to horseshoe crab blood, rFC, is equally effective in biomedical research, yet it has not been widely adopted due to financial incentives [31]. On the other hand, adoption of some alternatives, such as AquaBounty salmon, suffers from anti-GMO concerns [90]. 

These alternatives to wild harvest are not new; rFC was first developed in 1995 [91,92] and AquaBounty salmon were first developed in 1989 [32,33]. From an industrial perspective, both alternatives are competitors with wild harvesting industries, which supply local coastal communities with thousands of jobs. However, the decline of wild resources poses an equally negative impact to those fisheries, as well as the entire downstream consumer markets that utilize these products and which the alternative sources can maintain. The cost of adoption of synthetic alternatives is far less than the cost of depleting wild resources beyond recovery.

There appears to be growing public readiness for plant and cellular-based alternatives; global sales of meat-alternatives have increased 8% annually since 2010 [93]. While not yet as successful as terrestrial meat alternatives, plant- and cell-based seafood may ultimately grow faster as a category. Underpinning causes for this include the rapidly growing global unmet demand for seafood, the potential collapse of important fisheries, consumer awareness around environmental challenges, consumer fears around pollutants such as mercury or microplastics that bio-accumulate in wild fish, and significant investment interest from countries in Asia that view alternative meats as a means of ensuring better food stability [94]. This transition will likely be facilitated by applying lessons from the development, commercialization, and rapid demand for plant-based substitutes for meat.

The engineering and gene editing tools upon which interventions rely are dependent on (1) *in silico* genomic research, (2) in vitro cellular technologies, and (3) transition from in vitro to in vivo (made possible through advanced reproductive techniques to turn in vitro cells into embryos or the in vivo gene editing of embryos or adults). Intervention tools are well established in model species for biomedical and functional genomic research (i.e., fruit flies, zebrafish, mice), but are also advancing for agricultural species. For application to wildlife conservation, these require development and innovation, but are achievable short-term, with laboratory examples of success already established for some terrestrial species (e.g., transgenic chestnut trees immune to fungal blight [95], successful gene drive trials in mosquitoes [44]). The real barrier to intervention is having enough knowledge to construct phased-approaches that advance from the lab to the field effectively and safely and that obtain the intended benefits of intervention while avoiding unintended consequences. Predicting, avoiding, and setting in place mitigation strategies for unintended consequences is essential for successful genetic rescue applications, from seemingly nondisruptive translocations or facilitated adaptation guided by genetic insight to the genomic interventions that spur most concerns; hence, the importance of building genomic resources and ecological studies, for which both genetic insight and intervention can expound.

The use of genetic engineering for conservation is mired by concern among the public and the scientific community over unintended or negative consequences. Marine applications pose an even higher burden of proof due to the shared nature of marine ecosystems, making stakeholder engagement critical to building guidelines for standard practices. Therefore, efforts should be targeted at evaluating and mitigating risks, working with regulatory bodies, and engaging the public. For example, the technical challenges for developing genomic biocontrols of marine invasive and irruptive species are significant, but not impossible. However, genetic biocontrol is controversial and there exists concern that the unintended effects of biological control are a threat to the environment and would potentially cause nontarget species or populations to become extinct, highlighted by the current developmental shift from self-propagating gene drives to self-limiting versions [96]. In addition, the regulatory environment is justifiably cautious, constantly changing, and varies between countries.

## 5. Discussion

**Figure 3:**
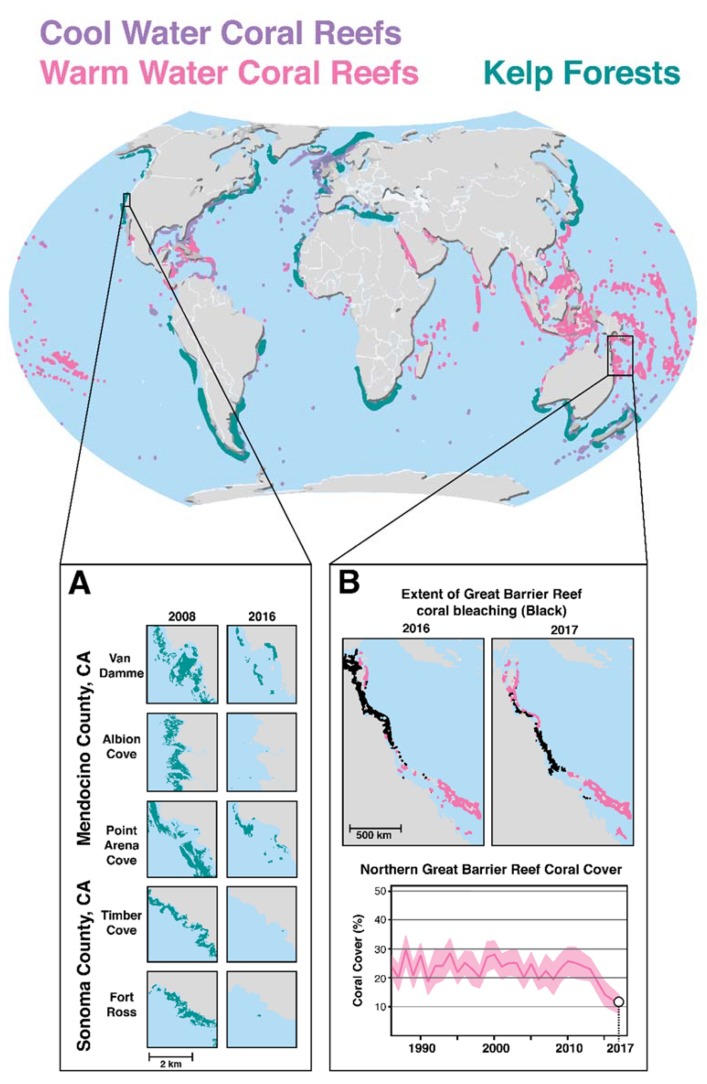
Global Distributions of Coral Reefs and Kelp Forests. (**A**) Presents northern California kelp forest loss from 2008 to 2016. (**B**) Presents the first consecutive severe bleaching event in Great Barrier Reef Survey history from 2016 and 2017. Sources of maps and graphs: [104,105], California Department of Fish and Wildlife Marine Management Team Aerial Surveys, and Australian Institute of Marine Science (AIMS).

The decline of ocean species destroys more than the evolutionary potential of biodiversity; it degrades human livelihoods as well. Societies are economically reliant upon natural resources, both biotic and abiotic, which can only be provided by functional ecosystems. The Stockholm Resilience Center has identified strong links between biodiversity loss and poverty and human suffering. For example, over 35% of global food production is dependent on pollination. The loss of pollinating species could present a major food security challenge [97]. Nearly 40% of the world’s population resides in coastal communities directly benefiting or reliant on the marine resources of coral reefs and kelp forests, two autogenetic ecosystems that cumulatively support the highest levels of marine biodiversity (and subsequently support important fisheries). Both are rapidly declining due to climate change exacerbated threats (Figure 3). Fisheries in the United States provide 1.6 million jobs and $208 billion per annum [98]. In the most widely cited study, Pimentel et al. [99] estimated that invasive species in the United States cause economic losses totaling $120 billion per year. 

The compounding effects of climate change stand to simultaneously reduce the value of the fisheries industry and increase the severity of invasive species problems, causing a dual front of economic loss, which will be felt more deeply in poorer coastal nations. The global community cannot be dominated by the dialogue of rich nations when considering all options to prevent biodiversity losses, a point we assert given the historic precedence of repeated health and economic harm caused by extreme biotechnology regulation in developing nations which were inspired by US and European anti-GMO lobbyists, rather than sound science or balanced discourse [100,101,102,103].

Even with powerful enhancement via genetic insight, current conservation strategies are not sufficient to address all environmental threats, and given the international complexities of oceans, no single sector, application, or method can address the multi-faceted nature of these challenges. International genetic rescue focused programs, working groups, and consortia will be necessary to achieve specific conservation goals. Augmenting traditional strategies with genetic insight and the appropriate genomic intervention, where beneficial, can ultimately produce synergistic conservation impacts. Though some may caution that genomic solutions should be a last resort, with careful and thorough development, early deployment of genomic intervention in certain situations will produce significantly higher conservation gains as opposed to postponing the intervention until no other tools can help yield success. 

When considering the options, there are three essential questions to ask: When should humankind intervene in a natural evolutionary process? Oceans are shared environments, and technological intervention should be developed in a manner that reflects the values and needs of the communities that depend upon and are in relationship with shared marine ecosystems. Moreover, what is the cost/benefit analysis of each solution? Equally important, what is the cost and risk of inaction? Comparing and contrasting alternative approaches may allow decision makers and the public to understand that not using genetic technologies may have equally, if not larger, negative consequences for marine ecosystems and society as a whole.

With ocean resources it is nearly impossible for a nation to form conservation policies that will not impact other nations—tracking of 1648 individuals of 14 Pacific species found that per year, these 14 species alone visit and utilize resources within the jurisdictions of 86% of Pacific nations, with many individuals visiting dozens of national jurisdictions each year [106]. Those essential questions, therefore, need to be addressed by international dialogue, as well as internally so that the discourse is not driven by particular cultural biases. Information needs to be presented without agenda, in order for nations to make independent domestic policy decisions and to enact effective genomic conservation solutions.

Building public trust and engaging feedback will be essential elements of conservation efforts involving genomic technologies, especially on a global scale. These processes will depend upon relationship building to earn the respect and trust of the public. Genuinely incorporating stakeholders into the decision-making process requires the design of mechanisms for input, which in turn necessitate transparency in decision-making. Responsible development of genetic rescue strategies will therefore depend upon an exploration of the underlying values that are motivating the project and shaping its design. For example, how decision makers relate to their natural environment will form the ethical justification for many of these interventions. Whether the ecosystem under consideration is perceived to have utilitarian value (i.e., valued for its benefit to humans) versus intrinsic value (i.e., value is independent of humans) could impact the resources and care available to save that ecosystem [107].

The following are the recommendations for stakeholder engagement and genomic program development/deployment arrived at during the Ocean Genomics Horizon Scan compilation:

1. Weighing Risks and Benefits: Genetic rescue program developers should conduct a thorough review of both the intended outcomes and the potential benefits of applying genetic insight or genomic intervention, as well as the risks and potential consequences. Questions to consider include: Have other more established interventions failed? Do other interventions, such as the application of antibiotics or pesticides, have potentially worse environmental consequences? Is this intervention the most efficacious, lasting, and least risky solution to an environmental problem, not merely an equivalent or novel approach?
2. Transparency: Genetic rescue program developers in collaboration with conservation managers should proactively identify and inform key stakeholders in the early stages of technology development. This includes partners from the private sector (particularly biotech firms), social science experts, public sector partners, international and research organizations, religious and ethical organizations, NGOs, and local communities. This should involve objective discussions with the public about the risks and potential impacts of proposed environmental solutions and also subjective discussions about values.
3. Procedures: Genetic rescue program developers should conduct systematic and data-driven reviews of recommended best practices. These should include surveys of recent intentional environmental release of organisms (including biocontrol, re-introductions, and translocations) into natural environments and studies of the resulting long-term effects.
4. Tests: Genetic rescue programs should first field test genomic technologies in contained environments to minimize unintended environmental consequences. These should be simple enough to be cost-effective, yet complex enough to sufficiently mimic natural ecosystems to yield useful data on the efficacy of developing technologies. Suitable test environments will be especially important for marine environments.
5. Predictions: Genetic rescue programs should employ computational models, particularly as they grow more sophisticated with time, to predict the long-term effects on ecosystems and highlight potential failures of specific applications of genetic technologies.
6. Measures: Genetic rescue scientists should employ standard metrics for measuring safety and efficacy at appropriate environmental scales agreed upon in collaboration with regulators, so that direct comparisons of data sets can be made, and so universal analytic tools can be developed and useful to final-stage decision making.
7. Protocols: Genetic rescue program developers should understand the regulatory approval processes and jurisdictions that currently guide the transition of new technologies from the lab to field trials, and should be prepared to help guide new policies when needed.
8. Public-Private Partnerships: Genetic rescue programs should collaborate with philanthropists, NGOs, agencies, and community groups in the implementation of these technologies to evaluate risk, to address controversies, and to gain critical stakeholder support. These groups can convene conversations, commission studies, identify priorities, and connect with civil society to identify safeguards.
9. Remediation: Genetic rescue program developers should prepare for the possibility of failure. In the event of unintended consequences, will it be possible to regain control of the organism? Possibilities for this include the creation of self-limiting gene drives.
10. Learnings: Genetic rescue program scientists and conservation managers should continuously monitor introduced organisms and the relevant environment once the technology has been deployed. Since these technologies remain new, these findings should be shared so that researchers learn of the successes as well as failures. Ultimately, transparency will allow successful intervention techniques to be more rapidly adopted globally.

Innovations in genetic insight and genomic intervention technologies will diversify and strengthen the pool of strategies for ocean conservation to provide targeted responses to difficult and urgent needs [108,109]. Responsible development necessitates engaging diverse viewpoints and expertise to steer the course. Policy often lags behind technology innovation. However, it is not too soon to anticipate high potential applications for genomic technologies in ocean conservation and begin the necessary preliminary discourse now. 

The goal should be to iteratively craft a set of guidelines that clearly communicate the social and ethical priorities of all affected societies, and articulate the full range of standards that genomic intervention must meet to be considered for environmental release. Such a framework would open the door to many new, powerful, and potentially less intrusive approaches to long-term environmental restoration and health. 

However, functional and effective guidelines need to be created through an iterative process, inclusive of bioethical, sociopolitical, and cultural considerations and values. Therefore, regulatory and governance frameworks should be developed and tested in concert with the technologies through projects and case studies. Such case studies are underway, and are building the foundation for assessments at the global level, as exemplified both by the diverse contributors of the Ocean Genomics Horizon Scan, from which the content of this paper is extracted, as well as the International Union for the Conservation of Nature’s recently published assessment of the prospects for biotechnology in conservation [54]. Both these reports, among others addressing biotechnology for conservation, are emerging at a pivotal time in world history, as global governments have finally acknowledged the biodiversity crisis and the need for swift and effective conservation action, as outlined in the summary of the United Nations [110]. With global awareness and consilience for urgent conservation action mounting in parallel to rapid biotechnological developments, now is the time to deploy ready genetic insight applications and develop the iterative framework for the beneficial deployment of genomic interventions.

## Figures and Tables

**Table 1:** List of some of the world’s genomics initiatives.

Genomic Initiative	Taxonomic Focus	Target	Status
Earth Biogenome Project (EBP)	All known eukaryote species	1,500,000	In Process
Darwin Tree of Life	All known UK eukaryote species	66,000	In Process
Vertebrate Genomes Project (VGP)	All known vertebrate species	70,000	In-process
G10k	One species of each vertebrate genus	10,000	Transitioned to VGP
B10K	All known bird species	10,000	In-process
Bat1k	All known bat species	1300	In-process
Global Invertebrate Genomics Alliance (GIGA)	Marine invertebrates	7000	In-process
i5k	Arthropods, primarily insects	5000	In-process
1000 Fungal Genomes Project	Fungal species	1000	In-process
